# Non/Low-Caloric Artificial Sweeteners and Gut Microbiome: From Perturbed Species to Mechanisms

**DOI:** 10.3390/metabo14100544

**Published:** 2024-10-11

**Authors:** Jiahao Feng, Jingya Peng, Yun-Chung Hsiao, Chih-Wei Liu, Yifei Yang, Haoduo Zhao, Taylor Teitelbaum, Xueying Wang, Kun Lu

**Affiliations:** Department of Environmental Sciences and Engineering, Gillings School of Global Public Health, University of North Carolina at Chapel Hill, Chapel Hill, NC 27599, USA

**Keywords:** artificial sweetener, gut microbiome, microbiome–host interaction, exposure, metabolism

## Abstract

Background: Non/low-caloric artificial sweeteners (NAS) are recognized as chemical additives substituting sugars to avoid caloric intake and subsequent sugar-derived diseases such as diabetes and hyperglycemia. Six NAS have been claimed safe and are authorized by the US Food and Drug Administration (FDA) for public use, with acceptable daily intake information available: aspartame, acesulfame-K, saccharin, sucralose, neotame, and advantame. However, the impacts of NAS on the gut microbiome have raised potential concerns, since sporadic research revealed NAS-induced microbial changes in the gastrointestinal tracts and alterations in the microbiome–host interactive metabolism. Methods: Given the fact that the gut microbiome influences kaleidoscopic physiological functions in host health, this review aimed to decipher the impacts of NAS on the gut microbiome by implementing a comprehensive two-stage literature analysis based on each NAS. Results: This review documented disturbed microbiomes due to NAS exposure to a maximal resolution of species level using taxonomic clustering analysis, and recorded metabolism alterations involved in gut microbiome–host interactions. Conclusions: The results elucidated that specific NAS exhibited discrepant impacts on the gut microbiome, even though overlapping on the genera and species were identified. Some NAS caused glucose tolerance impairment in the host, but the key metabolites and their underlying mechanisms were different. Furthermore, this review embodied the challenges and future directions of current NAS–gut microbiome research to inspire advanced examination of the NAS exposure–gut microbiome–host metabolism axis.

## 1. Introduction

Over the past few decades, the global population has been facing the considerable health threats of obesity and cardiovascular diseases associated with high sugar consumption [1,2,3,4,5]. Non/low-caloric artificial sweeteners (NAS) became a remarkable dietary sugar replacement for combating the global prevalence of obesity and hyperglycemia [6]. NAS are a category of food additives utilized in food products, which are thought to bring a sweet taste and health benefits by avoiding the substantial energy content and carbohydrate intake from table sugar [7]. The U.S. Food and Drug Administration (FDA) has authorized six NAS with provided information on their acceptable daily intake: aspartame, acesulfame-K, saccharin, sucralose, neotame, and advantame. These six authorized sweeteners are claimed to be safe for the general population under certain conditions of use by the FDA, with support from scientific research [8,9]. Contradictorily, scientific research has revealed that NAS consumption was associated with multiple disease outcomes, for instance, liver cancer and chronic liver diseases [10,11,12], urinary tract cancer [13], kidney injury [14], and cardiovascular diseases [14,15,16,17,18]. In the meantime, more and more emerging evidence has highlighted that NAS might have complicated impacts on the gut microbiota [3,19,20]. However, the specific impacts of NAS on the gut microbiota are possibly underestimated when evaluating the safety and applicability of NAS.

Humans harbor diverse and dynamic microbial communities [21,22]. The gut microbiota is a collection of endogenous microorganisms that symbiotically inhabit the digestive tract [23]. Colonization of the gut microbiota begins in the proximal gastrointestinal tract, starting in the stomach and ending with diverse microorganisms in the distal gastrointestinal tract or the colon [24]. A healthy gut microbiota is typically characterized by high taxonomic diversity, extensive microbial gene richness, and a stable core of microbial species. The gut microbiota is generally composed of six dominant phyla: Firmicutes, Bacteroidetes, Actinobacteria, Proteobacteria, Fusobacteria, and Verrucomicrobia. Of these, Firmicutes and Bacteroidetes are the most prevalent [25]. Moreover, the gut microbiota plays a significant role in numerous physiological processes, such as immune system maintenance [25,26,27,28], drug metabolism [29,30,31], and biotransformation [32,33]. Previous research has shown that NAS such as saccharin and aspartame traverse the human gastrointestinal tract either undigested, indicated by their existence in excrement such as feces and urine, or digested, indicated by the detection of their secondary metabolites [34,35,36,37,38]. Taken together, consumption of NAS can induce their exposure to intestinal microbiota. 

We have found fragmented information illustrating the interaction between the gut microbiome and NAS exposure, and limited research was available to evaluate the toxicity of NAS on the gut microbiome in vivo. Notably, there is a lack of knowledge to comprehensively summarize the affected gut microbiome species and underlying host–microbe interactive metabolic mechanisms. These two components were essential for revealing the toxicology of NAS on the gut microbiome and for investigating the reciprocal influence in the NAS–gut microbiome–host axis. Consequently, the purpose of this review is to summarize the current understanding of the impacts of NAS on the gut microbiome, and to provide a guideline for future research on determining the gut microbiome–NAS interaction and gut health outcomes in the host. This review leveraged a two-stage exploratory review, primarily focused on uncovering the affected gut microbiome, followed by an illustration of the interactive metabolic mechanisms, including the metabolism pathways and signature metabolites, altered in the host as a response to NAS. Taxonomy clustering analysis was implemented to classify the biological relationships of NAS-disturbed species. This review also documented the current challenges and future directions in NAS–microbiome–host-related research. Meaningfully, this review provides fundamental information to advance the understanding of NAS interference on the gut microbiome, and contributes to decision-making on the safety of NAS in the future. 

## 2. Materials and Methods

This review is based on a two-tier comprehensive literature analysis on the PubMed and Google Scholar databases. The purpose of the literature analysis was to investigate the associations among the use of low/no-calorie sweeteners (NAS), changes in the composition of the gastrointestinal microorganisms, and the subsequent metabolism alterations in the host. The aim of the literature search was to maximize the information input of current academic literature that examined the impact of the six officially recognized NAS on the gut microbiota, which is commonly referred to as the gut or intestinal “bacteria”, “microbiome”, “microbiota”, “microbes”, “microflora”, or “microorganisms”. Furthermore, the investigation encompassed the inclusion of several designations for sweetening agents, which were enumerated as follows: aspartame; sucralose, also recognized as Splenda; saccharin; acesulfame potassium, also identified as Acesulfame K or Ace-K; neotame; and advantame. A combination of phrases related to sweeteners and terms related to gut microbiota were input to the database generating the primary literature pool on 8 August 2024. The search encompassed articles of all categories, without imposing any limitations on the dates of publication, which generated 63 articles on aspartame, 104 articles on saccharin, 153 articles on sucralose, 45 articles on acesulfame potassium, 7 articles on neotame, and 4 articles on advantame. This included scholarly publications that have undergone peer review, as well as non-peer-reviewed articles such as research articles, reviews, conference abstracts, news articles, interviews, editorials and opinions, and book chapters. Neotame and advantame, as second-generation sweeteners approved more recently by the U.S. FDA—neotame in 2002 and advantame in 2014—have had a shorter timeframe for research and commercial use, potentially limiting the incentive for extensive study. Additionally, their structural similarities to aspartame, a more prevalent or contentious NAS, may have further deprioritized research on them.

**Figure 1:**
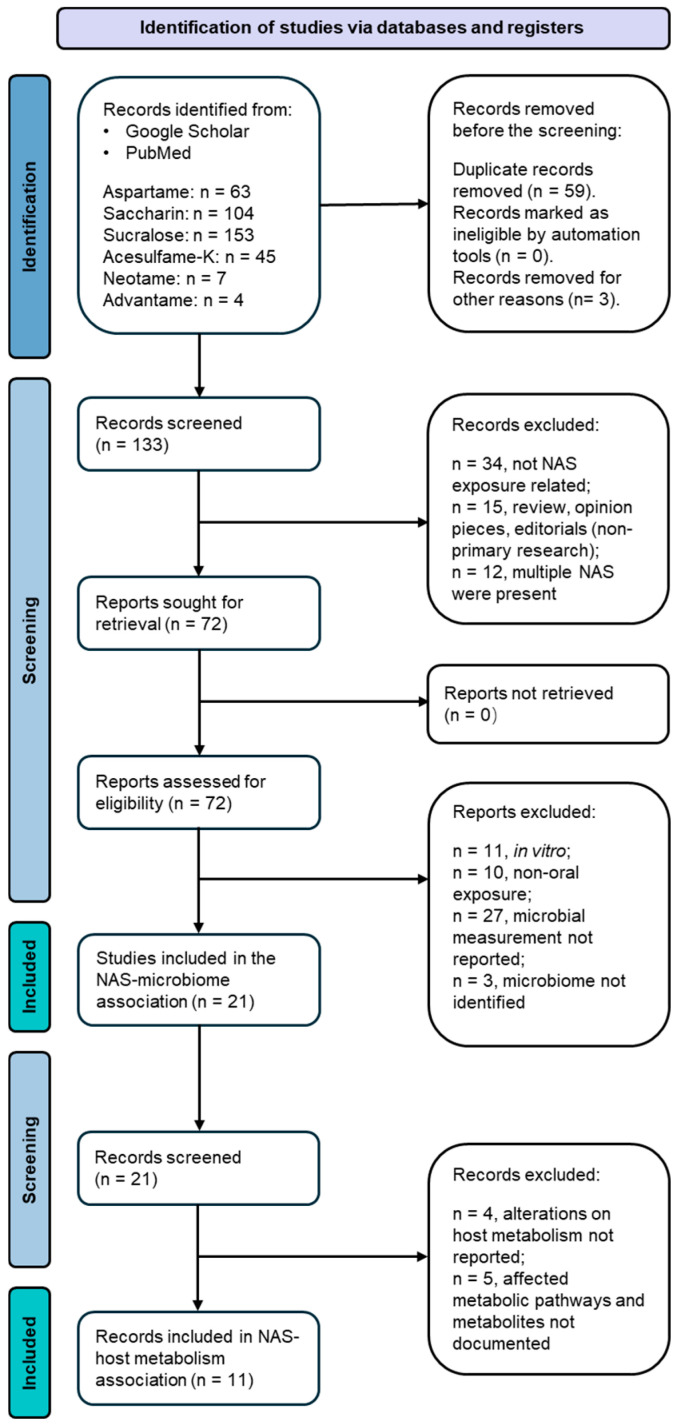
Flow diagram of the two-tier publication selection process for this review.

This review is composed of two consecutive screening stages(Figure 1). The first screening adopted a dual exclusion strategy. Initially, the first screening excluded repetitive articles in one or more databases, which generated 133 records for this review. Published abstracts from presentations and/or conferences were matched with full articles, where applicable, and the remaining abstracts were excluded in the search. These articles were screened for the following exclusion criteria: (a) records not related to NAS exposure; (b) records that were review articles, editorial and opinion pieces, or letters responding to recent publications in the field; and (c) records focusing on multiple NAS in a single experimental setting, multiple NAS categorized as a single variable, or co-existing NAS exposed to a single independent experimental subject. Beyond this exclusion, these articles were further screened for relevance based on the following inclusion criteria: (a) in vivo studies conducted in animals and humans (all in vitro studies were excluded); (b) oral exposure to NAS, which included diet intake or drinking water exposure; and (c) reported primary measurements of the microbiome in the gut. Following application of the defined screening criteria, 22 publications were identified as relevant primary research articles investigating the administration of NAS to animals or humans and the consequent disturbance to the gut microbiota. The empirical approaches and findings of these articles, encompassing variables such as sample size, participants, control groups, perturbation related to the gut microbiota, and analytical methods, have been extracted and classified based on the type of sweetening agent. The extracted gut microbiome information was input into the National Center for Biotechnology Information (NCBI) taxonomy database to conduct taxonomic cluster analysis, which displays a hierarchical clustering of bacterial taxa (phylum to species level) that are significantly impacted by different NAS. 

Based on the primary screening, we performed a secondary literature screening to collect available evidence on the association between the gut microbiota and metabolic consequences in the host. The screening aimed at documenting the altered metabolic pathways in the host and any metabolites or molecules that exhibited significant differences in the host. The exclusion criteria are listed as follows: (a) alterations in host metabolism were not reported using direct measurements derived from host biospecimen samples before or after NAS exposure; and (b) metabolites and affected metabolism pathways were not documented. This secondary screening resulted in 11 publications that served as the direct scientific evidence on integrating the triangle relationships among NAS exposure, the gut microbiome, and host metabolism. A zero match was found on advantame exposure in vivo in two-stage screening; thus, advantame was excluded in the meta-analysis. The empirical findings of these 11 articles, encompassing variables such as altered host metabolism pathways, key metabolites and their changing patterns in hosts before and after NAS exposure, and analytical methods, were extracted and classified based on the type of sweetening agent. 

## 3. Gut Microbiota Species Modulated by NAS Exposure 

### 3.1. Aspartame

The changes in the abundance of the gut microbiome affected by aspartame consumption were examined in rats after 8-week aspartame exposure in drinking water [39]. Even though the study introduced fat content in feeding as a second variable interacting with the first variable, the aspartame in water, the absolute bacterial analysis in the study concluded that *Clostridium leptum* was significantly higher (*p* < 0.05) in the low-fat feeding group fed with chow (12% kcal fat) and aspartame than in the control group fed with chow and water. *C. leptum* is known as a nitroreductase-producing bacterium, and its metabolism is associated with infective and chronic inflammatory bowel disease, including Crohn’s disease and ulcerative colitis [40,41,42]. *C. leptum* was also identified as a prominent bacterium that elicited quantitative differences between patients with type 2 diabetes and healthy humans [43], and showed a higher abundance in overweight adolescents [44]. Aspartame exposure attenuated the increase in abundance of *Clostridium cluster XI* in both the low-fat and high-fat diet groups [39]. However, treatment with aspartame in the high-fat diet (60% kcal fat) group resulted in the highest abundance of *Clostridium cluster XI*, *Enterobacteriaceae*, and *Roseburia* spp., and total bacteria, compared to the other groups fed either the low-fat or high-fat diets, with and without aspartame [39]. An increase of *Clostridium cluster XI* was witnessed in dietary habits involving intaking high levels of carbohydrate, fat, and protein, and its increase was positively correlated with the inflammation marker pro-inflammatory mucosal IL1-β concentration [45,46]. Interestingly, *Roseburia* spp. is an anaerobic rod-shape bacterium that can produce butyrate in the colon, and it contributes to multiple diseases, including inflammatory bowel disease, type 2 diabetes mellitus, and antiphospholipid syndrome [47], which exhibits a functional overlap with the *C. leptum* mentioned above. Nevertheless, the relative bacterial abundance analysis in the study conducted by Palmnäs et al. seemed to indicate that aspartame can also induce a decrease in *Lactobacillus* spp. and no changes in *Clostridium cluster I*, regardless of the fat content in feeding [39]. The changes of *Clostridium coccoides* and *Roseburia* seemed undifferentiable in the normal fat feeding group versus the aspartame-dosed normal fat feeding group, while such changes were notable when high-fat feeding was introduced, depicted as promoting the abundance of *Roseburia* and *Clostridium coccoides*. 

Acting not only in rats, aspartame consumption also altered the gut microbiome in humans, confirmed in a randomized-controlled trial encompassing 20 healthy adults administered aspartame for two weeks in a dose lower than the acceptable daily intake [48]. The results elucidated that top five glycemic responders’ aspartame exposure exhibited a positive association with *Bacteroides fragilis* and *Bacteroides acidifaciens*, and an inversely negative association with *Bacteroides coprocola* [48]. However, the same studies indicated that such changes in the gut microbiome could be customized in different hosts, with an emphasis that *Akkermensia muciniphila* increased significantly in human subjects showing the lowest glycemic response to aspartame exposure [48]. Further microbiome dissimilarities were displayed with the increase of *Clostridium* sp. *CAG:7* and *Tyzzerella* sp. *Marseille-P3062* and the decrease of *Alistipes obesi* and *Eubacterium* sp. *CAG:248* in the top glycemic responders compared to the bottom glycemic responders [48]. Among all aspartame-associated gut microbiomes in the study above, enterotoxigenic *Bacteroides fragilis* was recently reviewed to be possibly associated with colorectal cancer [49], while commensal bacterium *Bacteroides acidifaciens* was proven to participate in insulin protection in serum and β-oxidation in adipose tissues, protecting the host from diabetes and obesity [50,51]. *Akkermensia muciniphila*, a next-generation probiotic, has beneficial effects on glucose and lipid metabolism and the inflammatory response in humans, as well as on endotoxemia protection [52,53,54]. *Clostridium* sp. *CAG:7* is a purine-degrading prebiotic [55] and *Tyzzerella* sp. *Marseille-P3062* was positively associated with Crohn’s disease [56]. Altogether, aspartame exposure caused multiple changes in both probiotic and pathogenic bacteria and such bacterial consequences were customized in the host. 

### 3.2. Saccharin

A few studies were identified reporting that saccharin exposure can modify the abundance of the gut microbiome community. It was demonstrated that saccharin exposure in humans, at a dose of 0.18 g/day for 28 days, can cause significant changes in gut microbiota in experimental subjects who showed impairment of glucose tolerance [48]. Among the five top glycemic responders, saccharin exposure was positively associated with *Prevotella copri* [48], a species contributing to glucose homeostasis through enhancing bile acid metabolism and farnesoid X receptor (FXR) signaling [57], and negatively associated with *Bacterioides xylanisolvens*, a xylanolytic anaerobe known for its dietary fiber degradation and fermentation [58]. Meanwhile, the abundance of *Alistipes onderdonkii* was significantly higher during saccharin exposure but reduced to baseline levels in follow-up measurements [48]. The shift of *Alistipes onderdonkii*, an anaerobe exhibiting known pro-inflammatory activity which modulates the inflammatory response [59], might imply that saccharin exposure caused an inflammation response in the host, and activation of *Alistipes onderdonkii* might have healed the response in a short time. Furthermore, *Firmicutes CAG:102* showed an irreversible decrease in the long term after saccharin exposure [48]. Saccharin exposure also exhibited personalized effects on the gut microbiome in different humans. Top glycemic responders had a richer abundance of *Blautia* sp. *Marselle P2398* and *Clostridium* sp. *CAG:62* than bottom glycemic responders [48], in which *Blautia* sp. *Marselle P2398* was a marker reflecting major depressive disorder [60]. On the contrary, bottom glycemic responders had a higher abundance of *Bifidobacterium ruminantium*, *Clostridiales bacterium UBA 7739*, *Faecalibacterium prausnitzii*, and *Parabacteroides distasonis* than the top glycemic responders [48]. The same research team also evaluated fecal bacteria composition in mice exposed to saccharin in drinking water for 11 weeks, where they found over 40 operational taxonomic units were significantly altered in abundance using 16S RNA sequencing. At the strain level, *Bacteroides uniformis* were over-represented in the saccharin-exposed group compared with the control, while *Lactobacilluys Reuteri* were under-represented [48]. *Bacteroides uniformis* can ameliorate the metabolic and immunological dysfunction in obese mice induced by a high-fat diet [61,62], and its elevation may indicate their potential protection mechanisms via the gut microbiome to the host. Moreover, a randomized controlled trial revealed that probiotic *Lactobacilluys Reuteri* supplementation can increase the insulin sensitivity and bile acid deoxycholic acid in serum in type 2 diabetic patients [63]. Thus, decreased *Lactobacilluys Reuteri* may imply a decrease in insulin sensitivity as well as in bile acid metabolism. Results from shotgun metagenomic sequencing further exemplified the over-representation of *Bacteriodies vulgatus* and the under-representation of microbiome *Akkermansia muciniphila* in the saccharin-exposed group [7], which was coherent to the gut microbiome changes previously reported in patients with type 2 diabetes [64]. 

### 3.3. Sucralose

Sucralose, similar to saccharin, has been extensively documented for its alteration of the gut microbiome. Abou-Donia et al. reported that administration of Splenda (which contains sucralose) by oral gavage, at different concentrations of up to 1000 mg/kg for 12 weeks in rats, resulted in a significant decrease in beneficial gut bacteria [65]. Even at the lowest dose (100 mg/kg/d) the bacterial counts of *bifidobacterial, lactobacilli,* and *Bacteroides* were reduced by 36.9%, 39.1%, and 67.5%, respectively. Similarly, another study reported the alteration of 14 genera after exposing C57BL/6J to sucralose at 0.1 mg/mL in drinking water for six months, which were an increased abundance of *Turicibacteraceae turicibacter*, *Lachnospiraceae ruminococcus*, *Ruminococcaceae ruminococcus*, *Verrucomicrobiaceae akkermansia*, and unclassified members in the families *Clostridiaceae* and *Christensenellaceae*; and the decreased abundance of *Staphylococcaceae staphylococcus*, *Streptococcaceae streptococcus*, *Dehalobacteriaceae dehalobacterium*, *Lachnospiraceae anaerostipes*, *Lachnospiraceae roseburia*, and unassigned *Peptostreptococcaceae*, *Erysipelotrichaceae*, and *Order bacillales* [66]. However, these studies did not provide information on bacteria alterations at the strain level.

The impacts of sucralose on the gut microbiome exhibited solid evidence tracking back to the strain level. Human trials, by exposing healthy adults to sucralose under ADI for 28 days, revealed the alteration of three bacterial species, which were an increase of *Eubacterium CAG:352* and *Dorea longicatena*, and a decrease of *Oscillibacter ER4* [48]. However, sucralose exposure behaved differently on gut microbiome composition in different glycemic responders. *Bacteroides caccae*, *Bacteroides* sp. *Phil13*, and *Flavonifractor plautii* were three enriched species in the top glycemic responders, but were not shown in the bottom glycemic responders. The bottom glycemic responders accumulated more *Intestinimonas butriciproducens* than the top glycemic responders [48].

### 3.4. Neotame

Chi et al. reported that four-week neotame exposure in CD-1 mice facilitated the growth of two genera in the phylum *Bacteroidetes*, including *Bacteroides* and one undefined genus in *S24-7*, while significantly decreasing three genera in the family *Ruminococcaceae*, consisting of *Oscillospira*, *Rumniococcus*, and one undefined genus, and five genera in the family *Lachnospiraceae*, which contained *Blautia*, *Dorea*, *Ruminococcus*, and two undefined genera [67]. This is the only research focusing on neotame exposure and gut microbiome analysis.

### 3.5. Acesulfame Potassium

Acesulfame potassium can induce gut microbiome changes, but multiple variables may cause discrepancy in its gut microbiome changes. Bian et al. illustrated that acesulfame potassium induced sex-dependent alterations in gut microbiota [68]. In male mice treated with Ace-K via oral gavage at a dose of 37.5 mg/kg body weight/day, *Bacteroides* were highly increased, along with significant increases in two other genera, *Anaerostipes* and *Sutterella*. Notably, the four-week Ace-K treatment dramatically decreased the relative abundance of multiple genera in female mice, including *Lactobacillus*, *Clostridium*, an unassigned *Ruminococcaceae* genus, and an unassigned *Oxalobacteraceae* genus, and increased the abundance of *Mucispirillum*. Acesulfame potassium showed gut microbiome alteration in newborn mice when exposing their mother to acesulfame potassium. Olivier-Van Stichelen et al. illustrated that maternal exposure could induce defective *Akkermansia muciniphila* in newborns [69]. However, *Akkermansia muciniphila* showed no difference in growth when exposed to low or high acesulfame potassium in culture medium. 

A comprehensive summary of the documented alterations in the gut microbiome resulting from exposure to specific NAS, as detailed in Section 3.1, Section 3.2, Section 3.3, Section 3.4 and Section 3.5, is provided in Table 1.

## 4. Alterations of Metabolism in NAS–Microbiome–Host Interactions

### 4.1. Aspartame

Aspartame and its secondary products can reach to the colon, influencing the microbiota. Previous research elucidated that aspartame can be hydrolyzed in the intestines into phenylalanine, aspartame, and methanol. Aspartame consumption was associated with fasting hyperglycemia and impaired insulin tolerance in rats in a manner independent of fat intake in the diet, indicated by no difference in an oral glucose tolerance test and an elevated area under the curve for glucose in the insulin tolerance test in rats in both low- and high-fat diets [39]. Through an approach of administrating a high physiological insulin bolus into rats, the researchers clued that aspartame was able to reduce the capacity of clearing endogenous glucose, contributed by the mechanism of inducing an impairment of insulin-mediated suppression of net hepatic glucose output, than the deduction of peripheral insulin sensitivity [39]. The interlinks between aspartame and host health were presumed to be attributed to two gut microbiota changes, which were *Enterobacteriaceae* and *Clostridium cluster XI*, revealed in a high-fat diet in rats. The increase of *Enterobacteriaceae*, a member of the potentially harmful proteobacteria, produced gases and short-chain fatty acids that have been previously reported to be associated with inflammation and insulin resistance [84,85,86,87,88,89,90,91,92]. The decrease of the latter, as a member of the probiotic community, caused a disadvantageous condition of the community and may consequently have increased the amount of pathogenic bacteria in the gut microbiota [39]. 

The metabolites produced by the gut microbiota further entailed the putative mechanisms of how aspartame affected host health via gut microbiome-related physiological changes. Gut microbiome-derived metabolites represented end products of bacteria physiological activities and were the key intermediates bridging the host and the gut microbiome [33]. Aspartame exposure is associated with changes in acetate and butyrate [39]. The decrease of butyrate in the serum of the rats can be correlated with the observed decrease in *Clostridium cluster XI*, which are known as butyrate-producing bacteria. Another signature metabolite is propionate, which exhibited large elevations under the conditions of low-fat feeding as well as high-fat feeding. This change could be attributable to the increase of *Clostridium leptum* as it produces propionate when fermenting oligosaccharides [93,94].

Human studies reported that the modulation of the gut microbiome affected by aspartame exposure induced personalized but causative impacts on the glycemic response [48]. Such impacts were further demonstrated as significant metabolomic alterations in human plasma. Kynurenine, terephthalic acid, indole-3-acetate, and benzoate were four signature metabolites altered in the most sensitive responders to aspartame in the level of the glycemic response exposed to one-week of aspartame below ADI. Among them, kynurenine, indole-3-acetate, and benzoate were increased in the plasma, while the terephthalic acid was reduced. However, the detailed mechanism correlated with the metabolomic profiles and the microbiome changes affected by aspartame were not discussed. 

Previous research has mentioned that hosts who had experienced aspartame-induced gut microbiome dysbiosis developed alterations of multiple host metabolism pathways, which had the potential to be correlated with gut microbiome changes. Gluconeogenesis might be a potential mechanism through which the gut microbiome interfered with propionate production [39]. Moreover, pathways related to the urea cycle and its metabolites might be of prime consideration to understanding the interactions between gut microbiome dysbiosis and host health, as they were increased in the top aspartame responders to glycemic responses [48]. Along with these pathways, the negative association of the pathways in top glycemic responders, including phosphonate and phosphinate metabolism, flavin biosynthesis, L-histidine degradation, and L-proline degradation, may also be vital for further analysis on understanding the inhibition mechanisms of aspartame in aspartame–microbiome–host interactions [48]. 

### 4.2. Saccharin

The most direct existing evidence suggested that the mechanisms of the gut microbiome interfered with in humans who were exposed to saccharin and impaired glucose intolerance were related to 1) Uridine Monophosphate (UMP) biosynthesis and 2) glycolysis and glycan degradation [7]. The increase of UMP biosynthesis occurred simultaneously with the increase of *Prevotella copri* and shared the same pattern to an extent, which increased correspondingly along four timepoints: before exposure, Day 7 in exposure, Day 14 in exposure, and Day 28 after exposure. On the contrary, the decrease of glycolysis and glycan degradation occurred simultaneously with the decrease of *Bacteroides xylanisolvens*, and their changes were matched to each other in the exposure duration [48]. However, a study also reported that saccharin consumption also induced an increase in glycan degradation pathways and further annotation suggested that five Gram-positive and -negative bacteria species contributed to this increase, which were *Bacterioides fragilis*, *Bacteroides vulgatus*, *Parabacteroides distasonis*, *Staphylococcus aureus*, and *Providencia retteri* [7]. Such conflicts further explained that the gut microbiome was engaged in a scrimmage when exposed to saccharin. Moreover, the disturbance of metabolic pathways was displayed as a comprehensive interaction among multiple sub-pathways. The decrease of glycolysis and glycan degradation can be viewed as a weighted combined outcome of multiple sub-pathways, including homolactic fermentation, glycolysis I from glucose 6-phosphate, glycolysis II from fructose 6-phosphate, glycerol degradation to butanol, hexitol degradation, and Neu5Ac degradation [48]. Additionally, personalized differences were not neglectable between top glycemic responders and bottom glycemic responders in pathway changes. In top glycemic responders, five metabolomic pathways were significantly promoted, including (1) caprolactam degradation, (2) L-isoleucine biosynthesis II, (3) CDP-diacylglycerol biosynthesis, (4) glycerol degradation to 1.3-propanediol, and (5) mixed acid fermentation. In bottom glycemic responders, six metabolomic pathways were emphasized, which were (1) alanine, aspartame, and glutamate metabolism, (2) pentose phosphate metabolism, (3) L-serine and glycine biosynthesis I, (4) L-tryptophan biosynthesis, (5) histidine, purine, and pyrimidine biosynthesis, and (6) polyamine metabolism. Discrepancies in the affected pathways in human subjects further enlighten the complexity of deciphering the extent and magnitude to which specific bacteria strains contributed to pathway changes, thus increasing the difficulty in evaluating the interfering roles of the gut microbiome on their participation in these metabolic pathways. In the same study, serum metabolomic analysis revealed statistically significant changes in five metabolites: 4-hydroxybenzoate, benzoate, indoxyl sulfate, hexadecanedioic acid, and butyrate. Three of them, indoxyl sulfate, a metabolite related to vascular disease, 4-hydroxylbenzoate, and benzoate, increased during saccharin exposure, while hexadecanedioic acid decreased [48]. However, the study did not establish a detailed mechanism on how the gut microbiome may induce the above metabolomic changes that can result in host glucose tolerance impairment. 

### 4.3. Sucralose

There is clear evidence to support sucralose-induced physiological changes in the host via gut microbiome alteration. Sucralose increased the abundance of bacterial genes related to pro-inflammatory mediators, which featured the increase of genes related to LPS synthesis, flagella protein synthesis, and fimbriae synthesis as well as bacterial toxin and drug resistance genes [66]. In addition, fecal metabolomic analysis confirmed that sucralose altered quorum sensing signals, amino acids and derivatives, and bile acids. Furthermore, sucralose induced elevated pro-inflammatory gene expression in the liver, including matrix metalloproteinase 2 (MMP-2) and inducible nitric-oxide synthase (iNOS), that might be due to crosstalk along the gut–liver axis [66]. 

Another study depicted that sucralose exposure induced a significant increase in arginine biosynthesis, a significant decrease in mixed acid fermentation, and alteration in the Tricarboxylic Acid (TCA) cycle alongside the sucralose supplementation. Three other bacterial metabolomic pathways showed synergic decreases along the sucralose exposure timeline, including urate biosynthesis/inosine 5′-phosphate degradation, adenosine deoxyribonucleotide de novo biosynthesis, and guanosine nucleotide de novo biosynthesis [48]. The same study also evaluated changes in serum metabolites, in which they found increases in isocitrate, trans-aconitate, serine, N-acetylalanine, aspartate, quinolinate, 2-C-methyl-D-erythritol 4-phosphate, galactarate, and psicose; and decreases of pseudouridine, uric acid, and sebacic acid. The researchers performed enriched pathway analysis which highlighted the alteration of host pathways, including arginine biosynthesis and glutamine metabolism, aminoacyl-tRNA biosynthesis, and the TCA cycle. In alignment with the increased abundance of the TCA cycle in the gut microbiota, the quantitative measurements of two TCA intermediates, isocitrate and trans-aconitate, increased in the serum of human subjects after experiencing sucralose exposure, enlightened the connection between the gut microbiome and host via TCA cycle changes. Furthermore, the same study discovered that three plasma metabolites exhibited significant increases in the top glycemic responders compared to the bottom glycemic responders: beta-hydroxyisobutrate, cycteate, and serine [48]. These three metabolites may play an important role in inducing glycemic responses potentially linked to gut microbiome changes. 

### 4.4. Neotame

Neotame consumption has been proven to alter the metabolic pathways of gut microbiome. The available research revealed that streptomycin biosynthesis, amino acid metabolism, folate biosynthesis, and lipopolysaccharide biosynthesis were four pathways enriched in the neotame-treated microbiome, while seven other pathways were under-represented, including fatty acid metabolism, sporulation, benzoate degradation, carbohydrate metabolism, lipid metabolism, bacterial chemotaxis, and ABC transporters [67]. Additionally, the functional gene analysis further illustrated that the pyruvate-derived and succinate-derived butyrate fermentation pathways were perturbed by neotame exposure by alteration of the genes in enzyme production. In the pyruvate-derived butyrate fermentation pathway, four genes were reduced, which were the genes of acetyl-CoA C-acetyltransferase, 3-hydroxybutyryl-CoA dehydrogenase, 3-hydroxybutyryl-CoA dehydratase, and butyryl-CoA dehydrogenase. In addition, the genes of phosphate butyryl-transferase and butyrate kinase were increased. However, the genes of 4-hydroxybutyryl-CoA dehydratase, butyryl-CoA dehydrogenase, and acetate CoA-transferase were decreased in succinate-derived butyrate fermentation pathways [67].

### 4.5. Acesulfame Potassium

Acesulfame potassium induced carbohydrate metabolism changes in the gut microbiota, but exhibited sex-dependent features. The direct evidence, reported by Bian et al., claimed sex-specific alterations of functional genes in the gut microbiome exposed to acesulfame potassium [68]. In female mice, many of the key genes related to energy metabolism were decreased, consistent with the decrease of multiple microbiome bacteria in the female mice. These genes were involved in carbohydrate absorption and transportation, fermentation and degradation, polysaccharide hydrolysis, glycolysis, and the TCA cycle. There were no exceptions in that all these genes tended to be inhibited in the female mice, thus restricting the expression of multiple proteins such as glucose uptake proteins, lactose permease, sugar and D-allose transporters, different phosphotransferases, L-xylulokinase, α-amylase, and D-xylonolactonase. In contrast, the carbohydrate absorption and metabolism pathways were activated in male mice, in alignment with their increase in gut microbiome *Bacteroides*. The genes involved in carbohydrate metabolism and fermentation pathways, sugar and xylose transportation, glycolysis, and the TCA cycle were all increased. Altogether, acesulfame potassium might interfere with the gut microbiome–host interaction by inducing sex-specific gene alterations [68]. 

Furthermore, acesulfame potassium induced increasing gene abundance correlated to lipopolysaccharide synthesis (LPS synthesis) that might further increase the risk of inflammation in the host. Bian et al. demonstrated that genes involved in LPS synthesis also underwent sex-specific alterations after acesulfame potassium exposure. In female mice, some LPS synthesis-related genes as well as LPS-export genes were decreased, including UDP-glucose:(Heptosyl) LPS alpha-1,3-glucosyltransferase, ADP-L-glycero-D-manno-heptose 6-epimerase, amino-4-deoxy-L-arabinose transferase, UDP-D-GlcNAcA oxidase and UDP-GlcNAc3NAcA epimerase. Meanwhile, genes encoding flagella components were also increased. However, in male mice, only two LPS synthesis genes, which were glycosyltransferase and UDP-perosamine 4-acetyltransferase, and one bacterial toxin synthesis gene, which was thiol-activated cytolysin, were enriched. In addition, Bian et al. further demonstrated the changes in key fecal metabolites [68]. In female mice, three metabolites, which were D-lactic acid, succinic acid, and 2-oleoylglycerol, were significantly decreased. Conversely, two metabolites, pyruvic acid and cholic acid, were increased and one metabolite, deoxycholic acid, was decreased in the male mice after acesulfame potassium exposure. Even though the study did not correlate the LPS synthesis alteration and key fecal metabolites to the changes in the gut microbiome on the species level, changes in the bacterial genes supported by the fecal metabolites provided solid support for interlinking gut microbiome–host interaction mechanisms with LPS synthesis. 

A comprehensive overview of the documented metabolic alterations associated with specific NAS, as discussed in Section 4.1, Section 4.2, Section 4.3, Section 4.4 and Section 4.5, is provided in Table 2.

## 5. Challenges in Deciphering Underlying NAS–Gut Microbiome Mechanisms

It remains debatable if NAS modulations on the gut microbiome were due to direct interactions between NAS and the gut microbiome when NAS was orally administered. Scientific evidence has emerged revealing that each NAS exhibits unique absorption, distribution, metabolism, and excretion (ADME) patterns in organisms, and thus their kaleidoscopic alterations on the gut microbiome described in Section 3 can be the consequences of the host response in relation to their ADME differences. Clued by its unique chemical conformation, sucralose is a stable NAS that cannot be digested into monosaccharides or metabolized for energy. The substitution of hydroxyl groups in sucralose to chlorine in sucralose resisted sucralose from being cleaved by glycosidic enzymes that are capable of hydrolyzing sucrose and other carbohydrates. Combined with other evidence reported in toxicological studies targeting sucralose ADME among several species, not only in human [34], but also in mouse [36], rat [37], dog [35], and rabbit [36] studies, the biological fate of sucralose has been evaluated to be similar, depicted as low absorption and little to no metabolism in the host, regardless of species differences. Quantitatively, orally administered sucralose was proved to be excreted for 68.4% to 99% of the total dose in the feces, and only 2% to 26.5% was excreted in the urine [34,35,36,37,38], which illustrates that sucralose is unlikely to enter bodily circulation. Even though the high sucralose excretion percentage in the feces indicated that sucralose was barely absorbed or metabolized in the host, it can also serve as evidence that the majority of sucralose reached the large intestine and further implied that the gut microbiome was forced to be exposed directly to unmetabolized sucralose. Furthermore, a small portion of sucralose, varying from 2% to 35%, was absorbed [34,35,36,37,38], and its fate remains mysterious, even though sucralose-associated metabolites have been detected in urine, feces, and tissues [95]. Unlike sucralose, approximately 85% to 95% of administered saccharin was absorbed to the host plasma and was eventually eliminated unchanged in urine, as the principal method of plasma clearance [96]. Saccharin was absorbed by the gut lumen, where saccharin binds reversibly to plasma proteins and is distributed via the blood to the host body organs and eventually excreted in the urine [97,98,99,100,101]. However, in humans, the plasma concentration of saccharin and its time profile after oral dosage was shown to be complex, as its initial elimination was rapid during the first 10 h and then slowed down, and the slow phase was determined by prolonged absorption from the gastrointestinal tract, known as flip-flop kinetics [98]. This provided a novel insight that saccharin exposure is likely to induce long-term chronic toxicity via direct exposure to the gut microbiome, corresponding to its plasma clearance patterns, especially when active tubular transport, a primary mechanism of the renal elimination of saccharin, is saturated and causes excessive accumulation of saccharin [102]. Furthermore, research has consolidated that saccharin was excreted without undergoing metabolism in animals and humans [98], which increased the likelihood that saccharin impacted the gut microbiome via direct interference. Acesulfame potassium displayed similar absorption and excretion to saccharin, as the majority of acesulfame potassium via oral administration was absorbed rapidly and completely into the systematic circulation, and at least 82% of the absorbed acesulfame potassium was excreted in the urine within 24 h after consumption [34,35,36,37,96]. Aspartame has unique pharmacokinetics because it is quickly digested and hydrolyzed into methanol, phenylalanine, and aspartate in the gastrointestinal tract [103,104]. Aspartame is broken down in both the gastrointestinal lumen and inside intestinal mucosal cells by esterase and peptidases [103,104,105,106]. Thus, direct exposure of aspartame on the gut microbiome is unlikely to be the dominant mechanism [107]. Two second generation amino-acid based sweeteners, neotame and advantame, as two analogs of aspartame [108], may exhibit similar metabolism patterns as aspartame does in the gut microbiome–host interaction, and the impacts of their subsequent metabolites in the stomach and intestines may outweigh their direct exposure on the gut microbiome. However, for these two NAS, current knowledge is not adequate to depict whether their direct effects on the gut microbiome exist.

**Figure 2:**
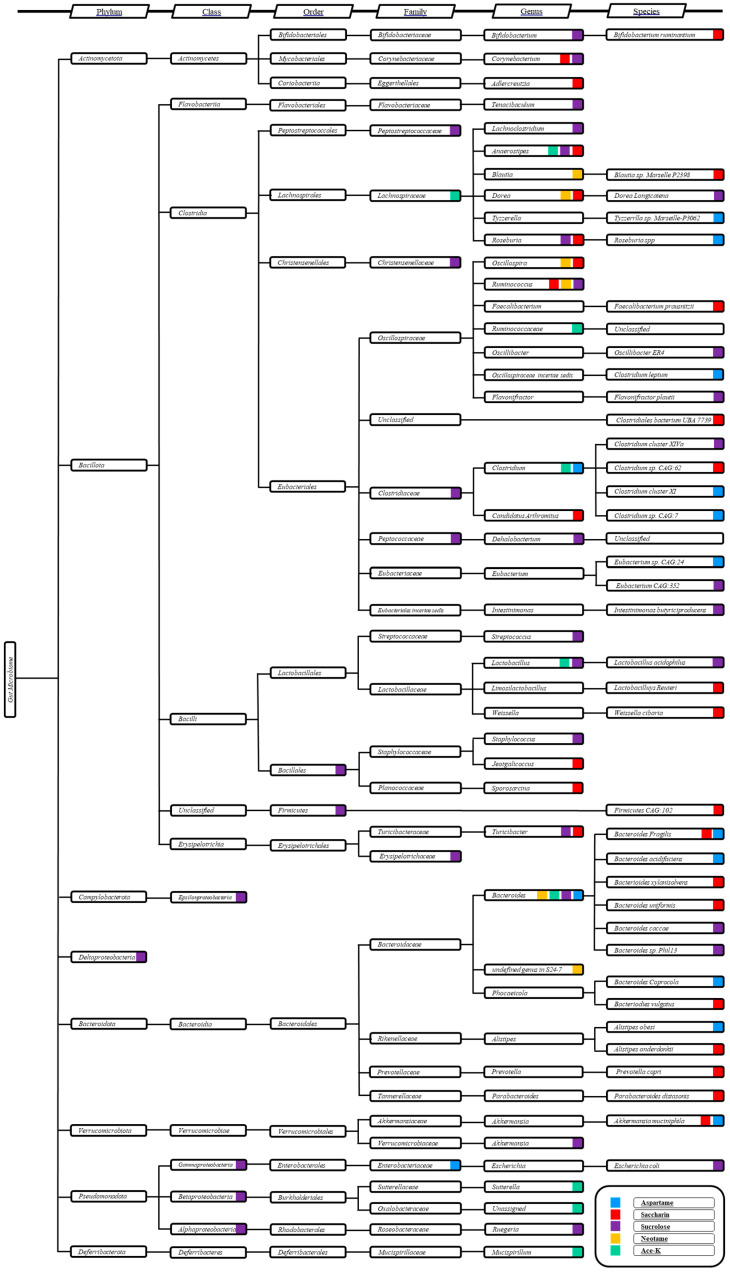
Taxonomic cluster analysis of the altered gut microbiome by specific NAS. The vertical axis represents the taxonomic hierarchy, with columns indicating the taxonomic rank from phylum to species. Color codes indicate the NAS associated with alterations in specific bacterial taxa.

The systematic effects of NAS exposure on the gut microbiome remain largely inscrutable. However, causality has been established through the utilization of germ-free rodent models, which unequivocally demonstrate that the gut microbiome is the principal driving force behind NAS-induced metabolomic perturbations and glucose intolerance in the host [48,109]. A comprehensive evaluation of gut microbiome succession in conjunction with NAS exposure is urgently warranted to unravel potential bacteria–bacteria interactions within the host gut microbiome in response to NAS. As inferred from extant literature, the alterations in gut microbiome composition are predominantly depicted through comparative analyses of microbiome profiles before and after NAS exposure. However, this approach fails to elucidate critical insights regarding the mechanisms by which the microbiome engages in NAS exposure. For instance, saccharin exposure has been observed to modulate the abundance of *Bacteroides uniformis* and *Lactobacillus reuteri* [48]; nevertheless, it remains equivocal whether these variations are the result of an abrupt shift due to NAS toxicity or a progressive transition driven by microbial metabolism in response to NAS assimilation. Therefore, understanding the dynamic transformations within the bacterial community is of paramount importance for accurately interpreting the long-term ramifications of NAS toxicity on the gut microbiome. Changes in specific species within the gut microbiome community may not provide an accurate assessment of NAS modulation, given the intrinsic nature of interspecies interactions. Among these, mutualistic effects and bacterial antagonisms represent two significant modes of bacterial interaction [110]. Mutualism describes scenarios wherein multiple bacterial species synergistically benefit from collaborative development and degradation of growth substrates, while antagonism underscores the competitive dynamics among bacterial species. Consequently, when interpreting a detectable alteration in a specific bacterial species following NAS exposure, multiple causative factors must be considered, including the following: (1) direct exposure to NAS (particularly if NAS is not metabolized in the stomach and intestines, such as with saccharin); (2) indirect interferences stemming from metabolomic alterations related to NAS absorption, distribution, metabolism, and excretion (if NAS is metabolized); (3) mutualistic effects involving dominant and other non-dominant bacterial species; and (4) competition with antagonistic species within the microbial community. The subset mechanisms underlying bacterial competition within the gut microbiome may further entail competition for beneficial substrates and competition for the optimal physiological environment. Validating which hypothesis predominantly contributes to NAS modulation of the gut microbiome is exceedingly challenging, as it necessitates an intricate analysis of community-level changes without compromising the resolution required to quantify the abundance of specific strains. The taxonomic clustering and mapping of bacterial species modulated by NAS exposure, as explored in this review (Figure 2), may provide the initial clues necessary to elucidate potential bacteria–bacteria cross-feeding mechanisms within the gut microbiome in response to NAS exposure.

Elucidating the endpoints of metabolic alterations induced by NAS remains a significant challenge, particularly in understanding how these sweeteners interfere with downstream gut microbiota-mediated physiological and biochemical processes in the host. Advances in metabolomics, especially through the use of high-resolution mass spectrometry (HRMS), have been pivotal in uncovering the complex metabolic chain reactions triggered by NAS exposure. HRMS-based metabolomics provides extraordinary precision in the quantification and identification of metabolites within the gut microbiome, offering detailed insights into how NAS can profoundly alter the microbial metabolic landscape. This high-resolution approach enables the detection of subtle yet critical changes in metabolic pathways that might otherwise be overlooked, such as the reprogramming of carbohydrate metabolism within the microbial community in response to NAS. The gut microbiota, when exposed to NAS, is forced to adapt by shifting from traditional sugar metabolism, like that of sucrose, to alternative pathways. This metabolic shift leads to significant changes in the production of short-chain fatty acids (SCFAs) and other vital metabolites [7,39]. HRMS has been instrumental in detecting these nuanced shifts, providing comprehensive metabolite profiles that help elucidate how the altered metabolic pathways influence key endpoints in host metabolism, including glycemic control, lipid metabolism, and insulin sensitivity—factors that are critical for maintaining overall metabolic health [111]. The ability of HRMS to reveal disruptions in these pathways is crucial, as it helps identify potential triggers of low-grade systemic inflammation, which may arise from metabolic byproducts that provoke immune responses, thereby contributing to conditions such as obesity, type 2 diabetes, and other metabolic disorders [112,113]. Despite the insights gained, the direct links between NAS-induced microbial changes and specific disease phenotypes in the host remain elusive, underscoring the need for further research. Integrating HRMS-based metabolomic pathway analysis with microbial gene expression and host metabolic data could provide a more comprehensive understanding of the intricate interactions between NAS, the gut microbiome, and host metabolism. This approach holds the potential to identify biomarkers of NAS-induced metabolic dysregulation and clarify the long-term health implications of these widely consumed sweeteners.

The physiological functions of SCFAs modulated by the gut microbiome to NAS exposure remain largely elusive, posing a critical challenge in understanding their broader impact on host health. SCFAs, primarily acetate, propionate, and butyrate, are key metabolic end products of gut microbiome activity and serve as essential mediators in host–microbiome interactions [33]. These fatty acids, produced through the fermentation of dietary fibers by gut bacteria, play vital roles in maintaining gut health, regulating immune responses, and influencing metabolic processes such as lipid metabolism and glucose homeostasis [114,115]. Emerging research suggests that NAS can alter gut microbiota composition, consequently affecting SCFA production, as documented in Table 2. Some studies indicate that NAS affect SCFA-producing bacteria, leading to alterations of SCFA in serum or in the excretes [7,39]. These alterations in SCFAs could have significant implications for host metabolism, as SCFAs are involved in promoting insulin sensitivity, regulating lipid metabolism, and modulating inflammation [112,116]. SCFAs also enter systemic circulation, where they influence physiological processes such as appetite regulation via the gut–brain axis, modulation of insulin sensitivity, and adipose tissue function [111,113]. The potential of NAS to disrupt these processes through altered SCFA production from the gut microbiome could profoundly impact metabolic health, contributing to conditions like obesity, type 2 diabetes, and cardiovascular disease [112]. Moreover, SCFAs play a crucial role in maintaining gut barrier integrity by enhancing tight junction protein expression and providing energy to colonocytes, preventing gut permeability and systemic inflammation [117,118]. The modulation of SCFAs by NAS through the gut microbiome has the potential to disrupt gut homeostasis, leading to further dysbiosis, increased gut permeability, and a heightened risk of inflammatory conditions such as inflammatory bowel disease (IBD) and irritable bowel syndrome (IBS) [119]. Additionally, NAS may influence SCFA production by altering fermentation processes among the gut microbiome, potentially favoring non-SCFA metabolites that exacerbate metabolic imbalances [39]. This shift in microbial metabolism could impair the host from utilizing SCFAs efficiently, undermining their beneficial effects on energy metabolism and immune modulation [115]. Thus, understanding the relationship between NAS consumption, gut microbiome alteration, SCFA production, and host metabolic outcomes is imperative for elucidating the long-term health implications of NAS and the mechanisms through which they may impact health and disease. 

Heterogeneity in the gut microbiome of organisms exposed to NAS can result in varied microbial alterations and distinct changes in metabolite profiles, leading to differential impacts on host physiology. Research by Suez et al. (2014) highlighted that NAS exposure leads to individualized effects, particularly in glycemic response impairment, which varies significantly across subjects [7]. This variability in response may be attributed to a complex interplay of extrinsic and intrinsic factors. Extrinsic factors such as diet composition [120], stress levels [121], and environmental exposures [122] play a crucial role in shaping the gut microbiome during NAS exposure, potentially leading to the promotion or suppression of specific microbial taxa. These alterations can influence metabolic pathways differently, thus contributing to the observed discrepancies in glycemic control. On the other hand, intrinsic factors, including individual differences in ADME [123], further complicate the host response to NAS. These intrinsic factors might result in the distinct bioavailability of NAS and its metabolites, thereby affecting the gut microbiome and metabolic outcomes in a highly personalized manner. Taken together, the heterogeneity between gut microbiome composition, metabolic processes, and host response underscores the need for innovative approaches to assess the impacts of NAS on human health in the future.

## 6. Limitation

Co-exposure to multiple NAS was not the primary focus of this review. It is undeniable that co-exposure research on multiple NAS in a strict experimental setting may also be beneficial to reveal the systematic alterations of the gut microbiome and host metabolism mechanism, which could reveal potential synergistic or antagonistic effects on gut microbiota and their subsequent impacts on metabolic health. The outcomes of these studies would be crucial for understanding the broader health implications of NAS consumption, especially in the context of metabolic syndrome and other related disorders [124]. However, without specified and detailed understanding of single NAS exposure and its consequences, the host metabolism alteration from simultaneous multiple NAS exposures in a laboratory setting cannot be attributed to the consequences of a single NAS, and thus it is not appropriate to categorize their outcomes as a single NAS in this review. Moreover, even though exposure to a NAS mixture is likely the dominant condition in human populations, NAS intake is always accompanied with other nutrients or ingredients, which undermines the credibility of whether the host metabolism alteration is induced specifically by NAS. As a consequence, the authors suspected that an investigation between the gut microbiome and multiple NAS exposure might not correspond to the collection of single NAS exposures to the gut microbiome. In the modern food industry, various NAS are commonly incorporated into foods and beverages, either individually or in combination, resulting in humans frequently consuming multiple NAS simultaneously [96]. The processing of different NAS during food production can lead to varied exposure levels and potentially synergistic or antagonistic effects within the gut microbiome, which complicates the understanding of the health impacts associated with NAS. Furthermore, when multiple NAS are consumed together, their combined effects on the gut microbiome and host metabolism become far more intricate, involving complex interactions between different microbial species and metabolic pathways [125]. These interactions due to co-exposure of multiple NAS can result in unpredictable changes in microbial composition and function, which subsequently influence the understanding of the gut microbiome–metabolic syndrome axis.

## Figures and Tables

**Table 1:** Summary of documented altered gut microbiomes affected by specific NAS.

NAS	Exposure	Altered Gut Microbiome *	Analytical Methods	Reference
Aspartame ADI: 50 mg/kg Sucrose equivalence: 200× 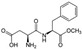	Rats, SD Normal rats (N = 10–12), 5 mg/kg/d, 8 weeks Obese rats (N = 10–12), 7 mg/kg/d, 8 weeks	Normal rats: *Clostridium leptum* ↑ Obese rats: *Clostridium cluster XI* ↓ *Enterobacteriaceae* ↑ *C. leptum* ↑ and *Roseburia* spp. ↑	Fecal DNA extraction + 16S rRNA sequencing + qRT-PCR analysis	(Palmnäs et al., 2014) [39]
	Humans, Exposed (N = 20), 0.24 g and 5.76 g glucose, 4 weeks	Top glycemic responders: *Bacteroides Fragilis* and *Bacteroides acidifaciens* ↑ *Bacteroides Coprocola* ↓ Bottom glycemic responders: *Akkenmensia muciniphila* ↑ Top compared to bottom responders: *Clostridium* sp. *CAG:7* ↑ *Tyzzerrlla* sp. *Marseille-P3062* ↑ *Alistipes obesi* and *Eubacterium* sp. *CAG:24* ↓	16S rRNA sequencing Illumina NextSeq platform	(Suez et al., 2022) [48]
	Humans, Exposed (N = 7), 1.7–33.2 mg/d, based on daily food records in four days Non-exposed (N = 24)	No significant changes	16S rRNA sequencing Length heterogeneity polymerase chain reaction (PCR) fingerprinting	(Frankenfeld et al., 2015) [70]
	Humans, Exposed (N = 15), 0.425 g/day, 14 days	No significant changes	16S rRNA sequencing Illumina MiSeq	(Ahmad et al., 2020) [71]
Saccharin ADI: 5 mg/kg Sucrose equivalence: 300× 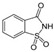	Humans, Exposed (N = 20), 0.18 g and 5.82 g glucose, 28 days	Top glycemic responders: Prevotella copri ↑ *Bacterioides xylanisolvens* ↓ *Alistipes onderdonkii* ↑ *Firmicutes CAG:102* ↓ Top compared to bottom responders: *Blautia* sp. *Marselle P2398* ↑ *Clostridium* sp. *CAG:62* ↑ *Bifidobacterium ruminantium* ↓ *Clostridiales bacterium UBA 7739* ↓ *Faecalibacterium prausnitzii* ↓ *Parabacteroides distasonis* ↓	16S rRNA sequencing Illumina NextSeq platform	(Suez et al., 2022) [48]
	Mice, C57BL/6 Exposed (N = 20), 3333 mg/kg/d, 11 weeks Control (N = 20)	*Bacteroides uniformis* ↑ *Lactobacilluys reuteri* ↓ *Bacteriodies vulgatus* ↑ *Akkermansia muciniphila* ↓	16S rRNA sequencing	(Suez et al., 2014) [7]
	Humans, (5 males and 2 females, aged 28–36) Exposed, 5 mg per kg of body weight for 5 days	*Bacteroides fragilis* ↑ *Weissella cibaria* ↑ *Candidatus arthromitus* ↓	16S rRNA sequencing	(Suez et al., 2014) [7]
	Mice, male, C57BL/6J (8 weeks old) 0.3 mg/mL in water for six months	After three-month consumption: *Anaerostipes* ↓ *Ruminococcus* ↓ *Sporosarcina* ↑ *Jeotgalicoccus* ↑ *Akkermansia* ↑ *Scillospira* and *Corynebacterium* ↑ After six-month consumption: *Ruminococcus* ↓ *Adlercreutzia* ↓ and *Dorea* ↓ *Corynebacterium* ↑, *Roseburia* ↑ and *Turicibacter* ↑.	16S rRNA gene sequencing	(Bian et al., 2017) [72]
	Dogs, female beagles 0.02% saccharin and eugenol, or 5% fiber blend plus 0.02% saccharin and eugenol for 10 days (N = 8)	No shifts in fecal microbial richness and diversity	16S rRNA gene sequencing	(Nogueira et al., 2019) [73]
	Mice, C57Bl\6J and Whole body T1R2-deficient mice, (eight-week-old) 250 mg/kg for 10 weeks	No alterations in microbial diversity or composition at any taxonomic level.	16S rRNA gene sequencing	(Serrano et al., 2021) [74]
	Humans, 18–45 years old (1) pulp filler/placebo (1000 mg/day 1) sodium saccharin (400 mg/day), (3) lactisole (670 mg/day), or (4) sodium saccharin (400 mg/day) + lactisole (670 mg/day) twice daily for 2 weeks	No alterations in microbial diversity or composition at any taxonomic level	16S rRNA gene sequencing	(Serrano et al., 2021) [74]
	Wistar rats, Exposed, 20 and 100 mg/kg body weight/day for 28 days	No effects on microbiome changes	16S rRNA gene sequencing	(Murali et al., 2022) [75]
Sucralose ADI: 15 mg/kg Sucrose equivalence: 600× 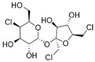	Mice, C57BL/6 J Exposed (N = 10), 9–22 mg/kg/d, 6 months Control (N = 10)	Turicibacteraceae Turicibacter ↑ *Lachnospiraceae ruminococcus* ↑ *Ruminococcaceae ruminococcus* ↑ *Verrucomicrobiaceae akkermansia* ↑ Unclassified members in Family Clostridiaceae↑*Christensenellaceae* ↑ *Staphylococcaceae Staphylococcus* ↓ *Streptococcaceae streptococcus* ↓ *Dehalobacteriaceae dehalobacterium* ↓ *Lachnospiraceae anaerostipes* ↓ *Lachnospiraceae roseburia* ↓ Unassigned *Peptostreptococcaceae* ↓ *Erysipelotrichaceae* ↓ *Bacillales* ↓	16S rRNA sequencing	(Bian, 2017) [66]
	Humans, Exposed (N = 20), 0.18 g and 5.82 g glucose, 28 days	Top glycemic responders: *Eubacterium CAG:352* ↑ *Dorea longicatena* ↑ *Oscillibacter ER4* ↓ Top compared to bottom responders: *Bacteroides caccae* ↑ *Bacteroides* sp. *Phil13* ↑ *Flavonifractor plautii* ↑ *Intestinimonas butriciproducens* ↓	16S rRNA sequencing Illumina NextSeq platform	(Suez et al., 2022) [48]
	Rats, SD Exposed (N = 10/group), Splenda 1.1, 3.3, 5.5 or 11 mg/kg/d, 12 weeks Control (N = 10)	*Bifidobacterial* ↓ *Lactobacilli* ↓ *Bacteroides* ↓	Culturing plates	(Abou-Donia et al., 2008) [65]
	Mice, C57Bl/6J mice (4 weeks old) Exposed (N = 8/group), sucralose 1.4 ± 0.1 mg/kg BW/day and 14.2 ± 2.2 mg/kg BW/day Control (N = 8)	*Clostridium cluster XIVa*↓	16S rRNA sequencing	(Uebanso et al., 2017) [76]
	Mice, SAMP1/YitFc (SAMP) Exposed(N = 5–7/group), 6-week supplementation of Splenda; ingredients: sucralose/maltodextrin, 1:99, *w*/*w*), 1.08 mg/mL; 3.5 mg/mL; 35 mg/mL	Five classes in *Proteobacteria* phylum ↑ (*Alphaproteobacteria*, *Betaproteobacteria*, *Gammaproteobacteria, Epsilonproteobacteria*, *Deltaproteobacteria*) *Escherichia coli* ↑	Culturing plates + 16S rRNA sequencing	(Rodriguez-Palacios et al., 2018) [77]
	Mice, C57BL/6 (5 weeks old) Exposed (N = 8/group), 8 weeks, sucralose (2.5%, *w*/*v*)	In chow-only mice: *Firmicutes* ↓, *Bacteroidetes* ↓, *Bifidobacterium* ↑ In high-fat-diet mice: *Firmicutes* ↑, *Bacteroidetes* ↓	16S rDNA sequencing	(Wang et al., 2018) [78]
	Mice, Pathogen-free (SPF) C57BL/6J, male, (28 days) Exposed (N = 8/group), 0.0003 g/mL, 0.003 mg/mL, 0.03 mg/mL, 0.3 mg/mL per day for 16 weeks	In jejunum: *Tenacibaculum* ↑, *Ruegeria* ↑ In ileum: *Staphylococcus* ↑, *Corynebacterium* ↑ In cecum: *Lachnoclostridium* ↓, *Lachnospiraceae UCG-006* ↓	16S rDNA sequencing	(Zheng et al., 2022) [79]
	Humans, 18–50 years old Exposed (N = 16), 780 mg sucralose/day for 7 days Control (N = 14)	No significant changes	16S rDNA sequencing	(Thomson et al., 2019) [80]
	Humans, 18–35 years old Exposed (N = 20/group), 48 mg Splenda/day for 10 weeks	*Lactobacillus acidophilus* ↓ *Blautia coccoides* ↑	16S rRNA sequencing Quantitative polymerase chain reaction (qPCR)	(Méndez-García et al., 2022) [81]
	Mice, C57BL/6J, 8 weeks old Exposed (N = 10), 0.1 mg/mL for 6 months	Lactobacillus ↓ Ruminococcus ↓	16S rDNA sequencing	(Chi et al., 2024) [82]
Acesulfame potassium ADI: 15 mg/kg Sucrose equivalence: 200× 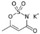	Mice, CD-1 Exposed (N = 5), 37.5 mg/kg/d, 4 weeks Control (N = 5)	Males: *Bacteroides* ↑; *Anaerostipes* ↑; *Sutterella* ↑ Females: *Mucispirillum* ↑, *Lactobacillus* ↓, *Clostridium* ↓, an unassigned *Ruminococcaceae* genus and an unassigned *Oxalobacteraceae* genus ↓	16S rRNA sequencing	(Bian, et al., 2017) [68]
	Mice, C57Bl/6J mice (4 weeks old) Exposed (N = 9/group), 15 mg/kg BW/day Control (N = 8)	No significant changes	16S rRNA sequencing	Uebanso et al., 2017) [76]
	Mice, C57BL/6J, (8 weeks old) Exposed 150 mg/kg b.w./day for 8 weeks	*Clostridiaceae* ↓ *Lachnospiraceae* ↓ *Ruminococcacea* ↓	16S rRNA sequencing	(Hanawa et al., 2021) [83]
	Humans, Exposed (N = 7), 1.7–33.2 mg/d, based on daily food records in four days Non-exposed (N = 24)	No significant changes	16S rRNA sequencing	(Frankenfeld et al., 2015) [70]
Neotame ADI: 18 mg/kg Sucrose equivalence: 7000–13,000× 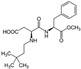	Mice, CD-1 Exposed (N = 5), 0.75 mg/kg/d, 4 weeks Control (N = 5)	*Bacteroidetes* including *Bacteroides* and one undefined genus in *S24-7* ↑ Three genera in the family *Ruminococcaceae*, consisting of *Oscillospira*, *Ruminococcus*, and one undefined genus, and five genera in family *Lachnospiraceae*, which contained *Blautia*, *Dorea*, *Ruminococcus*, and two undefined genera. ↓	16S rRNA sequencing	(Chi et al., 2018) [67]

* Note: The arrows (↑/↓) denote the modulation (increase/decrease) in the relative abundance of particular gut microbiota in response to specific NAS, as delineated in the referenced scholarly articles. These modulations are contingent upon the experimental frameworks and outcomes articulated in each study.

**Table 2:** Summary of documented altered metabolisms affected by specific NAS.

NAS	Altered Host Metabolism Pathways *	Key Metabolites Changes in Host **	Analytical Methods	Source
Aspartame ADI: 50 mg/kg Sucrose equivalence: 200× 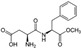	Fasting hyperglycemia and impaired insulin tolerance Pathways: Gluconeogenesis (?)	Acetate (?), butyrate ↓, propionate ↑	Serum metabolomic proton nuclear magnetic resonance spectroscopy (1H NMR)	(Palmnäs et al., 2014) [39]
	Altered glycemic response Pathways: Fatty acid degradation ↓ L-methionine biosynthesis ↓ Peptidoglycan biosynthesis ↓ Top compared to bottom responders: Pyrimidine nucleobases salvage ↑ L-omithine biosynthesis ↑ Heme biosynthesis ↑ Urea Cycle ↑ Phosphonate and phosphinate metabolism ↓ Flavin biosynthesis ↓ Pyridoxal 5′-phosphate biosynthesis and salvage ↓ L-histidine degradation ↓ L-proline biosynthesis ↓	Kynurenine ↑, terephthalic acid ↓, indole-3-acetate ↑, benzoate ↑	Serum metabolomic UPLC + Q-ToF mass spectrometry	(Suez et al., 2022) [48]
Saccharin ADI: Sucrose equivalence: 400× 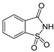	Altered glycemic response UMP biosynthesis ↑ glycolysis and glycan degradation ↓ homolactic fermentation ↓ glycolysis I from glucose 6-phosphate ↓ glycolysis II from fructose 6-phosphate ↓ glycerol degradation to butanol ↓ hexitol degradation ↓ Neu5Ac degradation ↓	4-hydroxybenzoate ↑, benzoate ↑, indoxyl sulfate ↑, hexadecanedioic acid ↓	Serum metabolomic UPLC + Q-ToF mass spectrometry	(Suez et al., 2022) [48]
	Induced glucose intolerance glycolysis and glycan degradation ↑	Propionate ↑ acetate ↑	Fecal metabolomic HPLC	(Suez et al., 2014) [7]
	Increased inflammation in the host by increasing the abundance of bacterial genes involved in elevating the pro-inflammatory mediators LPS synthesis ↑ Bacterial toxins ↑ Flagellar assembly protein ↑ Fimbrial protein ↑ Drug resistance ↑	Daidzein ↑ dihydrodaidzein ↑ O-desmethylangolensin ↑ Equol ↓ linoleoyl ethanolamide ↓ palmitoleoyl ethanolamide ↓ N,N-Dimethylsphingosine ↓ quinolinic acid ↑	Fecal metabolite analysis HPLC-Q-ToF	(Bian et al., 2017) [72]
	-	Acetate ↑, propionate ↑, butyrate ↑	Fecal metabolomic HPLC	(Serrano et al., 2021) [74]
	Altered amino acids, lipids, energy metabolism and bile acids in the plasma	-	Targeted MS-based metabolome profiling	(Murali et al., 2022) [75]
Sucralose ADI: 15 mg/kg Sucrose equivalence: 600× 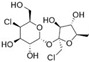	LPS synthesis ↑ Flagella protein synthesis ↑ Fimbriae synthesis ↑ Bacterial toxins and drug resistance genes ↑ Quorum sensing signals ↓ Amino acids and derivatives ↓ Bile acids (?)	N-butanoyl-l-homoserine lactone ↓, N-(3-oxo-hexanoyl)-homoserine lactone ↓, N-tetradecanoyl-L-homoserine lactone ↓, and N-pentadecanoyl-L-homoserine lactone ↓ L-tryptophan (Trp) ↑, quinolinic acid ↑, kynurenic acid ↓, and 2-aminomuconic acid ↑ L-tyrosine ↑, p-hydroxyphenylacetic acid ↓, and cinnamic acid ↓ 3-Oxo-4,6-choladienoic acid ↑, 3β,7α-dihydroxy-5-cholestenoate ↓, 3α,7β,12α-trihydroxyoxocholanyl-glycine ↓, and lithocholic acid/isoallolithocholic acid/allolithocholic acid/isolithocholic acid ↓	Fecal metabolomic HPLC-Q-ToF	(Bian, et al., 2017) [66]
	Arginine biosynthesis ↑ Mixed acid fermentation ↓ TCA cycle ↓ Urate biosynthesis/inosine 5′-phosphate degradation ↓ Adenosine deoxyribonucleotide de novo biosynthesis ↓ Guanosine nucleotide de novo biosynthesis ↓	Isocitrate ↑, trans-aconitate↑, serine ↑, N-acetylalanine ↑, aspartate ↑, quinolinate ↑, 2-C-methyl-D-erythritol 4-phosphate ↑, galactarate ↑, psicose ↑, pseudouridine ↓, uric acid ↓, and sebacic acid ↓	Serum metabolomic UPLC + Q-ToF mass spectrometry	(Suez et al., 2022) [48]
	Cholesterol–bile acid metabolism	Hepatic cholesterol ↑ cholic acid ↑, ratio of secondary bile acids (dehydrocholic acids (DCA) and lithocholic acid (LCA)) to primary bile acids (CA and CDCA) ↑	Liver/cecal metabolomic LC-MS	(Uebanso et al., 2017) [76]
	1. Richness of bile salt hydrolase gene (choloylglycine hydrolase) ↓, secondary bile acid synthesis pathway ↓ 2. Bile acid compositions and Farnesoid X Receptor (FXR) activation: (1)Ratios of various free bile acids and taurine-conjugated bile acids, including αMCA/TαMCA, ωMCA/TωMCA, CDCA/TCDCA and DCA/TDCA ↓, moderately for βMCA/TβMCA (*p* < 0.07) and CA/TCA (*p* < 0.06) ↓ (2)Expression of genes of Farnesoid X Receptor (FXR) signaling in livers, including *Shp*, *Cyp7a1*, *Cyp27a1*, and *Ntcp* ↓ 3. Altered hepatic cholesterol homeostasis Expressions of genes encoding three major cholesterol efflux transporters, including *Abca1*, *Abcg5*, and *Abcg8* ↑ Expression of genes associated with reverse cholesterol transport (RCT), including Ldlr and Scarb1 ↓ 4. Disrupted hepatic lipid homeostasis expression of two nuclear receptors, Srebp1c and Chrebp ↑ Acc1 gene and Cd36 gene ↑	Hepatic lipid ↑, ceramide ↑, hosphatidylethanolamines ↑, phosphatidylserines (PS) ↑, phosphatidylcholines (PC) ↑↓	Metabolomics and hepatic lipidomic UHPLC-ESI-TSQ Quantis triple quadrupole mass spectrometer	(Chi et al., 2024) [82]
Acesulfame potassium ADI: 15 mg/kg Sucrose equivalence: 200× 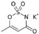	Female: Carbohydrate metabolism ↓ Lipopolysaccharide synthesis ↑ Male: Carbohydrate adsorption and metabolism ↑ Lipopolysaccharide synthesis ↑	Female: lactic acid ↓, succinic acid ↓, 2-Oleoylglycerol ↓ Male: pyruvic acid ↑, cholic acid ↑, deoxycholic acid ↓	Fecal metabolomic LC-MS	(Bian et al., 2017) [68]
Neotame ADI: 18 mg/kg Sucrose equivalence: 7000–13,000× 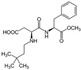	Streptomycin biosynthesis ↑ Amino acid metabolism ↑ Folate biosynthesis ↑ Lipopolysaccharide biosynthesis ↑ Fatty acid metabolism ↓ Sporulation ↓ Benzoate degradation ↓ Carbohydrate metabolism ↓ Lipid metabolism ↓ Bacterial chemotaxis ↓ ABC transporters ↓ Butyrate fermentation pathways	Malic acid ↓, mannose-6-phosphate ↓, 5-aminovaleric acid ↓and glyceric acid ↓, 1,3-dipalmitate ↑, 1-monopalmitin ↑, linoleic acid↑, stearic acid ↑, cholesterol ↑, campesterol ↑, stigmastanol ↑	Fecal metabolomic GC-MS	(Chi et al., 2018) [67]

* Note: The arrows (↑/↓) signify the upregulation or downregulation of metabolic pathways influenced by specific NAS, as detailed in the referenced studies. ** Note: The arrows (↑/↓) represent alterations (increases/decreases) in the levels of key metabolites detected in the host following exposure to specific NAS, as reported in the referenced studies and subject to the specific experimental conditions.

## Data Availability

Data sharing is not applicable.
